# Melatonin as an Adjuvant to Antiangiogenic Cancer Treatments

**DOI:** 10.3390/cancers13133263

**Published:** 2021-06-29

**Authors:** Alicia González, Carolina Alonso-González, Alicia González-González, Javier Menéndez-Menéndez, Samuel Cos, Carlos Martínez-Campa

**Affiliations:** Department of Physiology and Pharmacology, School of Medicine, University of Cantabria and Instituto de Investigación Valdecilla (IDIVAL), 39011 Santander, Spain; gonzalav@unican.es (A.G.); agonzalez.bq@gmail.com (A.G.-G.); javierrollo@movistar.es (J.M.-M.); martinezcm@unican.es (C.M.-C.)

**Keywords:** melatonin, angiogenesis, chemotherapy, radiotherapy, breast cancer, antiangiogenic actions

## Abstract

**Simple Summary:**

Chemo- and radiotherapy have an outstanding function in cancer treatment. In the last few years, in order to reduce the occurrence of adverse effects and to increase the efficacy of these treatments, there has been a growing interest in bringing together chemotherapeutic agents and ionizing radiation with other adjuvant therapies, antiangiogenic therapy being an example. Within the antitumor actions of melatonin is the inhibition of angiogenesis in diverse cancer types. In this review, we summarize present knowledge on the act of melatonin as a promising cancer cells sensitizer molecule to chemo- and radiotherapy through antiangiogenic actions.

**Abstract:**

Melatonin is a hormone with different functions, antitumor actions being one of the most studied. Among its antitumor mechanisms is its ability to inhibit angiogenesis. Melatonin shows antiangiogenic effects in several types of tumors. Combination of melatonin and chemotherapeutic agents have a synergistic effect inhibiting angiogenesis. One of the undesirable effects of chemotherapy is the induction of pro-angiogenic factors, whilst the addition of melatonin is able to overcome these undesirable effects. This protective effect of the pineal hormone against angiogenesis might be one of the mechanisms underlying its anticancer effect, explaining, at least in part, why melatonin administration increases the sensitivity of tumors to the inhibitory effects exerted by ordinary chemotherapeutic agents. Melatonin has the ability to turn cancer totally resistant to chemotherapeutic agents into a more sensitive chemotherapy state. Definitely, melatonin regulates the expression and/or activity of many factors involved in angiogenesis which levels are affected (either positively or negatively) by chemotherapeutic agents. In addition, the pineal hormone has been proposed as a radiosensitizer, increasing the oncostatic effects of radiation on tumor cells. This review serves as a synopsis of the interaction between melatonin and angiogenesis, and we will outline some antiangiogenic mechanisms through which melatonin sensitizes cancer cells to treatments, such as radiotherapy or chemotherapy.

## 1. Introduction: Melatonin, an Antitumor Hormone

Melatonin, the most important compound produced mainly by the pineal gland, exerts a relevant function in the modulation of cancer growth, particularly in mammary tumors, which present hormone-dependent growth. Many published studies carried out in animal models have shown a reduction in genesis and growth of breast cancer after treatment with melatonin, while pinealectomy has the opposite effect and normally increases tumor development [1,2]. Additionally, melatonin has also been shown to be a compound with a wide range of antitumor actions against many of the processes that favor cancer growth, such as cell proliferation, invasiveness, angiogenesis, or apoptosis in in vitro models with human breast cancer cells [3,4,5]. Melatonin synthesis in the pineal is controlled by the light-dark cycles so that light inhibits melatonin synthesis and, on the contrary, darkness allows its production and release into the blood. Consequently, one of the main characteristics of melatonin is its circadian rhythm with low blood levels throughout the day and large blood levels in the dark period.

Melatonin is an extremely lipophilic compound that can pass the circulatory barriers and reach high concentrations in the tumor and adipose tissue of the mammary gland [6]. Melatonin actions are mainly mediated through specific G-protein coupled membrane receptors (MT_1_, MT_2_) but also can readily pass through the cell or the nuclear membrane and bind to orphan receptors or can also interact with calmodulin, giving rise to non-receptor mediated effects [7]. Becker-Andre et al. (1994) described the activation of retinoic acid-related orphan receptors alpha (RORα) by melatonin [8]. Nevertheless, these results could not be confirmed later. However, it is very well known that melatonin can bind to calmodulin affecting intracellular calcium levels and modulating the transactivation of several transcription factors and nuclear receptors [9,10]. Other non-receptor mediated mechanisms involve actions of melatonin reducing oxidative damage [11], modulation of immune system [12], or the suppression of telomerase activity [13,14].

Melatonin actions have been demonstrated in different biological contexts. Melatonin has been related to synchronization of circadian rhythms, stimulation of immune system, antioxidant properties, antiestrogenic effects, oncostatic actions, etc. Within the latter, in the last few decades, many works have demonstrated the capacity of melatonin to inhibit tumor growth. These oncostatic actions take account through a variety of biological mechanisms: inhibition of synthesis of estrogens at the level of the gonads [15]; local melatonin antiestrogenic actions exerted directly on the tumor cell [16,17]; arrest of cell cycle; induction of differentiation and regulation of apoptosis [2,18]; activation of the immunity [19]; suppression of telomerase activity in cancer cells [13,14]; free radical scavenger and antioxidant effects [11]; suppression of tumor metabolism [20,21]; and reduction of angiogenesis [22,23,24]. This last mechanism, the development of new vessels, is a main stage in cancer growth. Since, among the multiple antitumor actions of melatonin is the ability to inhibit angiogenesis, the major aim of this review is to bring together all the information about the antiangiogenic mechanisms of melatonin and its implications as adjuvant to other cancer treatments. In this revision, we will firstly point out the importance of formation of new vessels in cancer development; secondly, we will underline how the interaction between melatonin and angiogenesis has been reported to justify the oncostatic actions of melatonin, and, lastly, we will summarize some antiangiogenic actions through which melatonin sensitizes malignant cells to radiation therapy or chemotherapeutic agents.

## 2. Angiogenic Factors and Cancer

Neovascularization refers to the formation of new blood vessels. There are different pathways comprising neovascularization; thus, while vasculogenesis indicates formation de novo of new blood vessels from endothelial precursors, angiogenesis involves the development of new vessels from a preexisting vascular network [25,26]. Angiogenesis occurs during embryonic development, bone morphogenesis, the menstrual cycle, and pregnancy [27,28]. Hypoxia is the main signal triggering the angiogenic response [25]. Moreover, angiogenesis is a characteristic of several pathologies, such as cancer [27]. From the physiological point of view, the endothelium has two states, quiescent and activated. In the quiescent state, the endothelial cells are covered by pericytes and smooth muscle cells, which form a stable vascular network through adhesion molecules, which are involved in the interaction between cells or with the extracellular matrix [29]. In this state, endothelial cells have abundant tight junctions with presence of integral membrane proteins (claudin, occludin, and transmembrane adhesive molecules JAMs) and cytoplasmic anchor proteins (ZO-1, ZO-2, ZO-4, and cingulin), providing a quiescent structural and functional integrity of the endothelium [30].

VEGF (vascular endothelial growth factor) is the main factor triggering angiogenesis. VEGF activates differentiation and proliferation of endothelial cells and formation of primitive vessels through the VEGF Flk1/KDR receptor (VEGFR-2) pathway. In addition to VEGF, the Angiopoietin 1 (Ang-1)/Angiopoietin Receptor (Tie) system has a fundamental role in remodeling, maturation, and stabilization of vessels [31]. However, in activated endothelium, Ang-2 binds to the Tie2 receptor, thus blocking Ang-1 binding [32]. As a consequence, the PI3K/AKT pathway is inhibited and endothelial quiescence is destabilized by dissociation of pericytes from endothelial cells. In the absence of VEGF, Ang-2 promotes regression of the vessel, whilst this destabilization, together with high levels of VEGF, promotes the angiogenic response [33]. Therefore, Ang-2 is a dynamic regulator of the Ang-Tie2 axis able to promote angiogenesis and inflammation [30].

In cancer, the formation of new vessels is essential for tumor cell survival and metastasis [34,35,36]. In tumors, lack of vessels, hypoxia, and nutrient deprivation trigger an angiogenic switch consisting in the expression of angiogenic promoters by tumor and stromal cells: VEGF, fibroblast growth factor (FGF), phosphogluconate dehydrogenase (PDG), lipoprotein A (LPA), and angiopoietins [25], allowing tumors to grow in size and to acquire metastatic potential [37]. Therefore, the formation of new vessels in tumors is a dynamic procedure in which a remodeling of the extracellular matrix, proliferation of endothelial cells, capillary differentiation, and anastomosis takes place [38].

The development of a functional vascular network in adult tissues is a process strictly controlled and regulated by growth factors that exert their action through specific endothelial receptor tyrosine kinases (RTKs), transmembrane proteins that promote the angiogenesis process. The three main families are: VEGFs/VEGFR, ephrins/ephrin receptors (EphR), and angiopoietin/Tie receptors [39,40]. Regarding the VEGFs family, VEGF-A is the main factor promoting hypoxia dependent angiogenesis in tumors [37,41]. VEGF is overexpressed in most human tumors and the grade of its expression correlates to vascular density of the tumor and is indicative of a worse prognosis. Hypoxia is a regulator of VEGF through hypoxia-inducible transcription factor (HIF) [42,43], formed by two subunits, α and β. The β subunit, also called ARNT, is a constitutive nuclear protein, whereas the α subunit level is modulated by the presence of oxygen [37,44]. VEGF transcription is triggered by HIF-1α and STAT3 (signal transducer activator of transcription 3), binding to the CBP/p300 coactivator on the VEGF promoter [45].

As mentioned above, the Angiopoietin/Tie system promotes endothelial cell survival, vessel remodeling, mural cell recruitment, and vessel maturation in embryogenesis and vascular maintenance [31,39,46,47]. Ang-1 and Ang-2 bind to the Tie2 receptor with similar affinity and activate it depending of the concentration [32,48]. The Ang/Tie2 system has a feedback regulation by which angiopoietins regulate the expression of their receptor in both physiological and pathological situations: an increase in the expression of angiopoietins leads to a decrease in the levels of the receptor by internalization and subsequent degradation [32,49]. Ang-1 is the main Tie2 agonist expressed in cancer cells. Ang-2 has been described as a Tie2 receptor antagonist, but it can also act as an agonist depending on the context. It is almost exclusively expressed in normal and tumor endothelial cells, where it is accumulated in the Weibel-Palade bodies [50]. Ang-2 promotes destabilization of the vasculature by antagonizing Ang-1-induced recruitment of mural cells to endothelial cells and enhances the vasculature response to angiogenic stimuli. The local cytokine environment largely determines the outcome of Ang-2 signaling. In the vasculature of tumors, when VEGF is present, Ang-2 induces migration, proliferation, and branching of blood vessels, while, in absence of VEGF, Ang-2 favor cell death and vascular regression [48]. These findings describe the antagonistic action of Ang-2 on the vasculature in contrast to the quiescent and anti-inflammatory effects of Ang-1 on endothelial cells.

**Figure 1:**
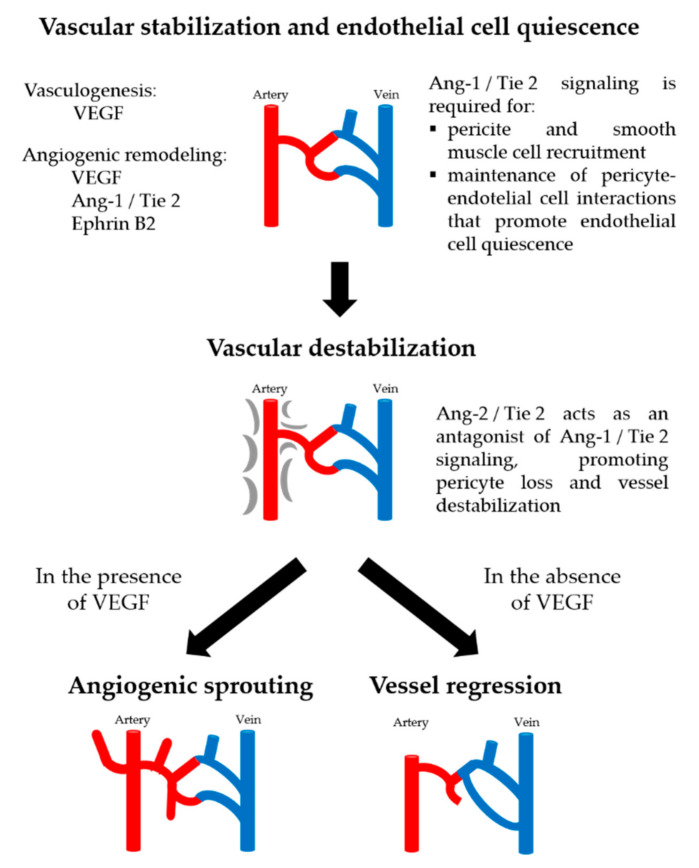
Vascular remodeling processes. Remodeling of the primary vessel is directed by VEGF, Ang-1, and Ephrin-B2 signaling pathways. Angiogenesis is characterized by endothelial cell activation and angiogenic sprouting, which require Ang-2-mediated vascular destabilization. In the presence of VEGF, this will lead to angiogenic sprouting, and, in the absence of VEGF, it will lead to vessel regression.

The global angiogenesis process is summarized in Figure 1: in the initial phases of development, vasculature formation occurs by vasculogenesis promoted by VEGF, thus forming a primitive vasculature; later, angiogenic remodeling occurs, thanks to VEGF, Ephrin B2, and Ang-1. While VEGF induces budding angiogenesis of endothelial cells, Ephrin B2 mediates bidirectional signals between adjacent cells and modulates cytoskeletal dynamics, affecting motility and adhesion [40]. In endothelial cells, Ang-1 is an agonist that induces the phosphorylation of Tie2, thus promoting the maturation, stability, and quiescence of the vessels [25,51], through endothelial cell survival and regulation of mural cell recruitment during physiological and pathological angiogenesis [25,48]. In contrast, Ang-2, acts in an autocrine manner on the endothelium, antagonizing the Ang-1 induced activation of Tie2 [29,48]. Furthermore, it can behave as a partial Tie2 agonist when Ang-1 is absent [29], or depending on the context [32]. Ang-2 interrupts the connections between the endothelium and perivascular cells promoting the destabilization of the pre-existing vessel [25], likely by blocking the constitutive stabilizing action of Ang-1 on endothelial cells. In absence of VEGF, regression and vascular death by apoptosis occurs [43]. However, in tumors, Ang-2 and VEGF induce destabilization of the vessels with subsequent neovascularization [34,36,40].

## 3. Melatonin Antiangiogenic Actions and Cancer

The first evidence that melatonin is an anti-angiogenic agent was obtained in metastatic patients who failed to respond to conventional chemotherapy and were treated with 20 mg/day of oral melatonin for at least two months. In those patients with minor answer or constant illness, a marked decrease in VEGF concentrations was observed, suggesting that melatonin acts as an anti-angiogenic molecule [52]. Soon after, the first study evaluating the effects of this indolamine in human umbilical vein endothelial cells (HUVEC) was published, demonstrating its ability to reduce Bcl-2 and to increase both Bax and p53 concentration, changes directly related to a diminution of cell proliferation, stimulation of apoptosis, and modulation of cell cycle length [53]. Recently, it has been demonstrated that melatonin’s ability to reduce the viability and angiogenesis of HUVECs might be explained since the pineal hormone downregulated the HIF 1α/ROS/VEGF axis [54].

After these works pointing to an anti-angiogenic action of the pineal hormone were published, many other studies have demonstrated this effect in many types of cancer. In breast tumor models, several results point to melatonin as an effective coadjuvant molecule for avoiding the angiogenesis around the tumor. Both VEGF gene expression and protein levels were reduced in HUVEC cells cocultured with MCF-7 (an estrogen-dependent mammary tumor cell line) when melatonin was added to the culture media. Moreover, melatonin blocked the cell proliferation stimulated by VEGF [23]. The anti-angiogenic action of melatonin might well be related to the estrogen availability in the tumor environment, since melatonin reduced the expression and activity of both classical aromatase promoter and aromatase promoter I.7 in HUVEC cells, indicating that melatonin reduced the production of estrogens, thus preventing carcinogenesis by interrupting the feedback between endothelial and tumor cells in hormone-dependent mammary tumor [22]. Additionally, melatonin reduced endothelial cells proliferation, invasion, migration, and tubulogenesis, mainly through the inhibition of VEGF, suggesting that the pineal hormone could be anticipated as a possible antiangiogenic molecule in the future treatments of breast cancer [24]. In HUVEC cells, melatonin can influence the phosphorylation of AKT (Ser473) but not ERK, thus diminishing vascular permeability [55].

Melatonin, at different concentrations, diminished the viability of MCF-7 and MDA-MB-231 (triple negative mammary tumor cell line) cells under normoxic conditions. The same concentrations of the indolamine, but under hypoxic conditions, decreased both HIF-1α and VEGF gene expression and protein concentrations. Likewise, in MCF-7 cells, melatonin reduced VEGF-C, VEGFR2, VEGFR3, MMP9, and angiogenin. However, in MDA-MB231 cells, the indolamine modified only VEGFR2, EGFR, and angiogenin. Specifically, in the absence of oxygen, EGFR protein expression notably augmented and melatonin was capable to diminish its levels. In vivo, melatonin reduced the expression of VEGFR2 protein in breast cancer of athymic nude mice. Finally, the pineal hormone decreased VEGF-C and VEGFR3 in MCF-7 cells [56]. Under hypoxic conditions, melatonin at 1 mM concentration reduced the viability of MCF-7 cells in parallel with a diminution of the concentration of VEGF, MMP9, VEGFR2, and VEGFR3 [57].

In mammary tumor, the antiangiogenic action of melatonin might be explained at least in part through regulation of pro- and anti-angiogenic miRNAs. Thus, in MDA-MB-231 cells, melatonin increased the levels of anti-metastatic miR-148b-3b and reduced the gene and protein expression of IGF-1R and VEGF, both in vivo and in vitro, causing a reduction action on migration and invasion of mammary cancer cells [58].

Rho-associate kinase protein-1(ROCK-1) is overexpressed in many types of cancer and is associated with tumor growth. ROCK-1 significantly stimulated cell migration and invasion by inhibiting PTEN and activating the PI3K/Akt pathway [59]. Rock inhibition is able to reduce many of the characteristics that play a role in cancer progression, especially by interfering with cell migration [60]. In addition, specific ROCK-1 inhibitors, such as Y27632, prevent metastasis in breast cancer [61]. When the action of melatonin in the programed cell death of mammary tumor was tested in in vitro and in vivo experiments, it was observed that melatonin and Y27632 reduced the expression of ROCK-1 and decreased cell viability, invasion, and migration either in cells capable of metastasizing (MCF-7) or in those that do not metastasize (MDA-MB-231) [62].

In estrogen related hypophysis-tumors, such as prolactinomas, melatonin administration showed an antiangiogenic effect by diminishing the levels of VEGF and MMP9, thus restraining the probability of development cells that are able to secrete prolactin [63].

The antiangiogenic actions of melatonin have also been tested in ovarian cancer models. The pineal hormone inhibited proliferation, angiogenesis, and metastasis in ovarian cancer cells. Interestingly, the expression of the MT1 receptor is higher in normal ovarian cells (IOSE36) than in cancerous cells (SK-OV3 and OVCAR-3), suggesting that melatonin induced an accumulation of cells in the G1 phase through inhibition of CDK2 and CDK4. Although there is some controversy about the effect of melatonin, since, in mice treated with melatonin at concentrations lower than 1 mM, the expression of HIF-1α, VEGF, and VEGFR2 were increased, in rats bearing endometrial implants, and treated with melatonin, there were lesser concentrations of VEGF, MMP9, and TIMP-2, less neovascularization, and increased rates of apoptosis [64].

The melatonin inhibitory effect on angiogenesis has been also found in hormone-dependent prostate cancer. In LNCaP and VCaP prostate cancer cell lines, melatonin inhibited proliferation through its binding to the MT_1_ receptor [65]. In the hormone-independent PC3 prostate cancer cell line, melatonin was able to inhibit sphingosine kinase 1 (SPHK1) and the Akt/glycogen synthase kinase-3β signaling, therefore impairing the stimulation of HIF-1α [66]. In male mice xenografted with LNCaP cells, melatonin augmented the expression of Nrf-2, diminished microvascular extent, and increased HIF-1α concentration [67]. Finally, back to PC3 cells, melatonin treatment induced the expression of miR-3195 and miR-374b and diminished the concentration of HIF-1α/1β, thus leading to suppression of VEGF production. This is the first time that someone suggests a posttranslational mechanism of melatonin concerning its action on angiogenesis [68].

The antiangiogenic actions of melatonin have been described in gastrointestinal cancers. Thus, in the pancreatic cancer cell line PANC-1, melatonin at pharmacological doses lowered both the expression and protein levels of VEGF provoked by CoCl2, likely through suppression of accumulation of HIF-1α [69]. In addition, in PANC-1 cells cocultured with HUVECs, melatonin at 1 µM and 1 mM reduced both HUVEC cell proliferation and migration through inhibition of secreted VEGF to the coculture medium. Additionally, melatonin also inhibited the proliferation of PANC-1 cells themselves [70].

In human gastric cancer, melatonin showed anti-angiogenic effects by downregulating nuclear receptor RZR/RORγ either in vitro and in vivo. In SGC-7901 cells, melatonin impaired VEGF secretion and reduced SUMO-specific protease 1, HIF-1α, and VEGF in provoking the inhibition of tumor growth. In melatonin treated nude mice bearing gastric cancer tumors, VEGF protein expression was significantly reduced, as well as the mRNA and protein levels of RZR/ROR receptor, SENP1, HIF-1α, and VEGF [71]. In vivo, melatonin inhibited peritoneal metastasis of gastric cancer, decreased the levels of epithelial-to-mesenchymal protein markers (E-cadherin, Snail, and Slug), and inhibited NF-kB, indicating that melatonin attenuated pro-angiogenic factors production [72]. Mechanistically, melatonin blocked the AKT/MDM2 pathway, thus stimulating apoptosis and impairing angiogenesis, which ultimately prevented the progression of gastric cancer [73].

In colorectal LoVo cancerous cells, melatonin-induced apoptosis was accomplished by dephosphorylation and nuclear import of histone deacetylase 4 (HDAC4), leading to downregulation of calcium/calmodulin-dependent protein kinase type II alpha chain (CAMKIIα) [74]. In the si-PRNP-transfected colorectal cancerous cells, the pineal hormone provoked the production of intercellular superoxide and endoplasmic reticulum stress and apoptosis. Concerning angiogenesis, the indolamine inactivated FoxO1 and NF-kB, decreasing then the expression of endothelin-1 and its liberation from cancer cells [75].

The growth of the hepatocellular carcinoma (HCC) takes place, thanks to the liberation of VEGF [76]. In hepatocarcinoma HepG2 cells, melatonin 1 mM reduced IL-1β induced nuclear factor-kappa B (NF-κB) translocation and transcriptional activity, and it prevented cell invasiveness across negative regulation of MMP-9 gene expression and positive regulation of TIMP-1 [77]. A similar result has been described in the same HepG2 cell line, where melatonin exerted its anti-angiogenic effects repressing IL-β induced NF-kB translocation and transcriptional activation and diminished HIF-1α protein expression and STAT3 activity [78]. In this same cell line, the suppression of STAT3 signaling diminished phosphorylation of STAT3 and CBP/p300 conscription as a transcriptional convoluted in the VEGF promoter, justifying the negative effects of melatonin on VEGF [45]. There are evidences that m-TOR/Akt stimulates angiogenesis, and, therefore, inhibition of this signaling pathway has therapeutic implications in many cancers [64,79]. In mouse hepatoma H22 cells, phosphorylation of m-TOR and Akt were reduced by melatonin leading to augmented Beclin-1 expression that intensified autophagy [80]. In an in vivo model, Abdel-Mawla et al. [81] observed a reduction of VEGF and PCNA gene expression in mice with 2-nitropropane-induced hepatocellular cancer treated with 10 mg/kg of melatonin. The conclusion they reached is that hepatic cells were protected by melatonin against carcinogens through its antiproliferative and antiangiogenic functions.

Melatonin exerts anti-metastatic and anti-angiogenic effects in renal carcinoma. In Caki-1 and Achn renal carcinoma cells, melatonin inhibited MMP9 thorough a reduction of the NF-kB binding to the MMP9 promoter. In samples from patients, an opposite correlation among the concentration of the MT1 receptor and MMP9 was established [82]. Renal carcinomas usually express high levels of ADAMTS1, which belongs to the ADAMTS family of metalloproteinases that promote tumor progression for its involvement in cell proliferation, adhesion, migration, and angiogenesis [83]. Melatonin, through induction of miR-let-7f and miR-181d, inhibits ADAMTS1 expression and triggers ADAMTS1 degradation by ubiquitin/proteasome [84]. Additionally, melatonin capacity to block tumor angiogenesis was defined in mice. The molecular mechanism resides in a decrease of the HIF-1α protein inside the tumor area, inhibiting the blood vessel formation inside the developing tumors without binding to the MT1 [85].

The anti-angiogenic actions of the pineal hormone were also described in several other kinds of tumors. Melatonin inhibited the proliferation, migration, and sprouting of the tertiary branching in chorioallantoic membrane and mesentery of Dalton’s lymphoma-bearing mice. In addition, the indolamine was capable to restore anti-angiogenic related genes, such as tissue inhibitor of metalloproteases 3 (TIMP-3), reducing, at the same time, the expression of VEGF, VEGFR, and FGF [86]. In glioblastoma stem-like cells, melatonin inhibited the pro-angiogenic EZH2-NOTCHI signaling pathway [87]. In squamous cell carcinoma SCC9 cell line, melatonin significantly inhibited the actions of HIF-1α and VEGF, in addition to the post-metastatic genes, such as ROCK-1, and attenuated MMP-9 expression and activity by lessening histone acetylation. Additionally, melatonin reduced the phosphorylation of ERK1/2 [88]. As already described in other types of cancer, melatonin decreased the growth and development of B16F10 melanoma cells by downregulating the pro-angiogenic PI3K/Akt/TOR pathway [89]. Likewise, in oral cancer cells, the pineal hormone diminished both proliferation and apoptosis by interfering with the ROS-dependent Akt signaling and ERKs, thus inhibiting angiogenesis [90].

## 4. Melatonin Antiangiogenic Actions and Chemotherapy

The oncostatic actions of melatonin have been reported not only for estrogen-responsive breast tumors but also for many other kinds of cancers, such as ovary cancer, gastric cancer, pancreatic ductal carcinoma, leukemia, non-small lung carcinoma, colorectal cancer, or cervical carcinoma [1,91,92,93]. As said above, melatonin inhibits angiogenesis through regulation of multiple cytokines and growth factors synthesized and released by breast cancerous cells in the proximity of the tumor, thus promoting cellular proliferation and tumor progression. One of the main objectives of the present review is to summarize the current knowledge about the interrelationship between melatonin and chemotherapy. In most cases, combination of melatonin and chemotherapeutic agents have a synergistic effect on angiogenesis inhibition, but it has also been reported that chemotherapy treatments can induce pro-angiogenic factors and importantly, the concomitant addition of melatonin overcomes this undesirable effect. Melatonin can transform cancers totally resistant to chemotherapeutic drugs to a sensitive chemotherapy state, at least in part by modulation of both expression and/or activity of many factors involved in angiogenesis in which levels are affected (either positively or negatively) by chemotherapeutic agents.

VEGF is the most active angiogenic factor since it is a highly specific mitogen for endothelial cells inducing their proliferation, migration, and new vessel formation, triggered by its binding to tyrosine kinase receptors [94]. High blood levels of VEGF correlate to poor prognosis in cancer patients. In those who progressed on previous chemotherapy treatments, melatonin slowed down the tumor growth. In non-progressing melatonin treated patients, the concentrations of VEGF showed an important decline, whilst progressing patients did not have any significant decrease of VEGF levels [63].

Pitavastatin is a tumor-suppressor statin in rat mammary carcinogenesis, reducing average tumor volume and lengthened latency; however, compared to controls, the statin augmented tumor frequency. When melatonin was combined with the statin, tumor volume and frequency were diminished, whereas latency was further increased. Pitavastatin alone increased VEGF expression, being this undesirable effect totally reversed by melatonin [95]. The combination of melatonin and another statin, pravastatin, increased the ratio between high- and low-grade carcinomas, induced expression of caspase-3 and caspase-7, and significantly reduced VEGFR-2 expression [96].

IL-25, an inflammatory cytokine, stimulates apoptosis in tumor cell lines. In canine mammary cells, the combination of IL-25 and melatonin reduced cell viability and significantly increase the levels of apoptotic proteins, such as caspase-3; additionally, the levels of VEGF-A were reduced in both metastatic and non-metastatic cell lines treated simultaneously with both compounds [97]. Thymoquinone, obtained from the black seed oil of *Nigella sativa*, is effective inhibiting different cancer stages: initiation, proliferation, migration, and invasion. When the anti-proliferative activity of thymoquinone and melatonin was tested against mouse epithelial mammary cancer cell line (EMT6/P), a synergistic anticancer effect of both molecules was detected. The combination therapy induced apoptosis, extensive necrosis and a marked decreased of VEGF expression in tumor sections [98].

Melatonin has also been tested in combination with the therapeutic cancer HPV-16 E7 DNA vaccine (human papillomavirus) against the syngeneic TC-1 tumor model (C57BL/6 mouse tumor model). The administration of the vaccine adjuvanted with melatonin significantly stimulated HPV16 E7 cytotoxicity and triggered IFN-γ and TNF-α responses, the growth of tumors was slowed down and the survival time of mice was lengthened. Further in vivo experiments demonstrated that melatonin decreased the levels of IL-10 and VEGF in the tumor microenvironment of vaccinated mice [99].

A new strategy combining melatonin and a ketogenic diet was assayed in parental (EMT6/P), cisplatin resistance (EMT6/CPR), and vincristine resistance (EMT6/VCR/R) murine breast cancer cells. The results obtained showed an important synergistic effect against cisplatin- and vincristine-resistant breast cancer cells xenotransplanted in mice. Additionally, a significant depletion in VEGF protein levels was found in the three cell lines, whereas a marked inhibition of PI3K and AKT was observed in sensitive and cisplatin-resistant cells [100].

In most cases, tumor growth depends on VEGF release; in parallel, hypoxia in the tumor microenvironment leads to intracellular stabilization of HIF-1α. In prostate PC-3 cancer cells undergoing hypoxia, melatonin blocked the expression of HIF-1α, the activation of Akt, and the production of VEGF. The effect of melatonin seems to be mediated by sphingosine kinase 1, a recently discovered regulator of HIF-1α, as corroborated by the use of a specific inhibitor of sphingosine kinase [66]. In hepatocellular carcinoma, stabilization of HIF-1α is accompanied by stabilization of signal transducer and activator of transcription (STAT3); in human HepG2 liver cancer cells, melatonin and Stattic (a selective STAT3 inhibitor) showed a synergistic effect on STAT3, HIF-1α, and VEGF expression levels by impairing the binding of the STAT3, HIF-1α, and CBP/p300 transcriptional complex to the VEGF promoter, thus decreasing its transcriptional activation [45].

The serine and threonine kinase Akt has physiological roles in nearly every organ system, but its dysfunction often is associated with many pathological settings, including cancer. Usually, the canonical pathway leading to Akt activation is initiated by the stimulation of receptor tyrosine kinases (RTKs) or G-coupled receptors (GPCRs), leading to activation of one or more isoforms of the class I PI3K family which, in turn, results in phosphorylation of Akt [101]. The PI3K/Akt/mTOR pathway plays a fundamental role in carcinogenesis, since its activation stimulates cell proliferation, promotes survival, enhances motility, and induces angiogenesis. The mechanisms responsible for this dysregulation include abnormal activation of receptors located upstream of PI3K, mutations in the gene encoding for the catalytic subunit of PI3K, such as amplifications or gain of function mutations, and/or loss of function of the tumor suppressor gene PTEN through deletion, mutation, or epigenetic silencing [102].

The combination of melatonin with chemotherapy not always proved to be effective in Akt inactivation: in SK-N-MC cells, a model of Ewing sarcoma, there is a synergy in the antitumor effect when melatonin is combined with vincristine or with cyclophosphamide; the synergism is due to the potentiation of cell death, especially by activation of the extrinsic pathway of apoptosis. Caspase-3, -8, -9 and Bid are activated when melatonin is included in the treatment; however, other proteins, such as mitogen-activated protein kinase or Akt, were not regulated by these treatments [103]. However, in many other cases, melatonin regulates Akt. Thus, in estrogen responsive breast cancer cells, melatonin and vitamin D3 synergistically inhibited cell proliferation and apoptosis, with a nearly complete cell growth arrest. The cytostatic effects triggered by vitamin D3 and melatonin were dependent on TGFβ-1, as demonstrated, since the employment of a monoclonal anti-TGFβ-1 antibody completely abolish these effects. In addition, melatonin and vitamin D3 caused a significant reduction of Akt activation, leading to an increase of p53/MDM2 ratio [104].

Melatonin also synergized the effect of 5-fluorouracil in colon cancer cells, inhibiting cell proliferation, migration, and invasive potential. Melatonin targeted the NF-kB and PI3K/Akt/inducible nitric oxide synthase (iNOS) signal pathway. Importantly, the pineal hormone strongly inhibited the phosphorylation of both PI3K and Akt and provoked the translocation of NF-kB (p50/p65) from the nuclei to the cytoplasm, thus abrogating its recruitment to the iNOS promoter. The effect was also replicated in a xenograft mouse model [105].

In esophageal squamous cell carcinoma (ESCC), the effect of 5-fluorouracil was completely different: in this case, the chemotherapeutic agent induced activation of Erk and Akt in ESCC cells, both in vivo and in vitro. Importantly, the pineal hormone inhibited proliferation, migration, and invasion and reversed the activation of Erk and Akt pathways, thus enhancing cytotoxicity of 5-fluorouracil in vitro and in vivo [106]. In Cal-27 (a head and neck squamous cell carcinoma), xenograft mice, it has been described the activation of the Akt mammalian target of rapamycin (mTOR) signaling pathway. Treatment with mTOR inhibitor rapamycin shows a limited effectiveness since normally leads to chemoresistance. When rapamycin and melatonin were combined, the negative feedback loop pf S6K1 to Akt was blocked, decreasing cell viability, proliferation, and clonogenic capacity, but increasing cell death and cellular differentiation [79].

Another in vivo study linked doxorubicin resistance with the disruption of the circadian melatonin signal by light at night (LAN) in human breast cancer xenografts grown in nude rats. The animals treated with either vehicle or doxorubicin showed high levels of p-ERK1/2, whereas those exposed to LAN and supplemented with melatonin showed almost complete suppression of p-ERK1/2 expression. Additionally, elevated levels of total AKT and NF-kB, but not their phosphorylated forms, were observed in LAN xenografts from animals treated with either vehicle or doxorubicin but were suppressed in the LAN xenografts from the animals receiving the pineal hormone [107].

In vincristine resistant human oral cancer cells, melatonin induced apoptosis and autophagy. These mechanisms involved regulation of Akt, p38 and c-Jun N-terminal kinase, likely through the upregulation of microRNA-892a and microRNA-34b-5p, thus exerting antiproliferative, antitumor invasion and migration, and antiangiogenic effects [108].

In a model of angiogenesis in vitro, it was demonstrated that melatonin regulated the response of HUVECs to vinorelbine and docetaxel. Melatonin enhanced the inhibition that these chemotherapeutic agents exerted on several processes required for angiogenesis: cell proliferation and motility, migration capacity or ability to form vessels. Rather surprisingly, vinorelbine and docetaxel stimulated the expression of angiogenesis-related factors, such as VEGF-A, VEGF-B, VEGF-C, VEGFR-1, VEGFR-3, ANG-1, and/or ANG-2, and, importantly, melatonin counteracted these actions. Docetaxel enhanced the expression of some other factors related to angiogenesis: IGF-1, MMP14, JAG1, ANPEP, CXCL6, ERK1, ERK2, AKT1, and NOS3, whereas vinorelbine stimulated the expression of FIGF, FGFR3, CXCL6, CCL2, ERK1, ERK2, AKT1, NOS3, and MMP14. Once again, melatonin counteracted the pro-angiogenic effects of both agents. In an in vivo chick chorioallantoic membrane model of angiogenesis, melatonin concomitantly administered with chemotherapeutic agents effectively inhibited new vascularization. The pineal hormone potentiated the inhibition of p-AKT and p-ERK observed after treatment with vincristine and docetaxel, and it abolished the chemotherapeutics-dependent permeability observed in HUVECs, likely due to modification of the distribution of VE cadherin [109].

The effect of co-treatment with melatonin and doxorubicin was tested in MCF-7 (estrogen-dependent) and MDA-MB-231 (triple negative) breast cancer cells. In MCF-7 cells, doxorubicin inhibited cell proliferation and had no effect on cell migration, but, strikingly, exerted a stimulatory effect over the invasive potential. Melatonin co-treatment further inhibited MCF-7 proliferation, repressed migration, and counteracted the stimulatory effect of the anthracycline on the invasive potential. These actions were not observed in triple negative MDA-MB-231 cells, suggesting that the effectiveness of melatonin may depend on the expression of the estrogen receptor. When the gene expression profile of angiogenesis related genes was examined in MCF-7 cells, the results revealed some undesirable actions of doxorubicin since the treatment with this anthracycline stimulated the expression of some pro-angiogenic genes, such as TWIST1 (twist-related protein 1), PLG (plasminogen), ANG-2 (angiopoietin 2), IGF-1 (insulin grow factor 1), GLI1 (glioma associated oncogene homolog 1), and BIRC5 (survivin). Importantly, melatonin counteracted these effects, indicating an anti-angiogenic effect of the pineal hormone [110].

The circadian clock genes might play an important role in tumorigenesis since they regulate the expression of angiogenesis related genes. For instance, low expression of the clock gene PER2 has been observed in carcinogenesis and the development of cancer. PER2 knockdown increased the expression of VEGFA [111]. Other regulators of circadian rhythms, such as BMAL1, can promote or repress angiogenesis, depending on the cancer type and probably also depending on the stage of tumor development. Thus, in lung cancer and glioma cells, BMAL1 knockdown induced cancer cell invasion in parallel with activation of the PI3K/Akt/MMP2 pathway [112], whereas, in drug-resistant metastatic colorectal cancer, BMAL1 promotes expression of genes involved in angiogenesis and tumor progression. Specifically, VEGFA synthesis was increased in patients with poor clinical response to Bevacizumab and high levels of BMAL1 expression [113]. In MCF-7 cells, doxorubicin treatment induced the expression of CRY1, CRY2, CLOCK, BMAL1, BMAL2, and PER2, and melatonin significantly further enhanced this stimulatory effect. The unique exception was TIMELESS, in which mRNA levels were increased after treatment with doxorubicin and inhibited when melatonin was included in the treatment. Doxorubicin strongly stimulated miR-10b, a microRNA related to oncogenesis, since its expression is associated to angiogenesis, invasion, and metastasis. Importantly, when melatonin was included in the treatment, this stimulatory effect was abolished [110].

Survivin, a protein encoded by the BIRC5 gene that controls cell division, metastasis, and angiogenesis, is usually strongly expressed in most human cancers [114]. It has been described that survivin expression in tumor cells is linked to increased β-catenin protein levels and, consequently, increased expression of factors regulated by the β-catenin-Tcf/Lef transcriptional pathway, such as VEGF, and it resulted in induction of angiogenesis in a PI3K/Akt-β-catenin-Tcf/Lef-dependent manner. In the chicken chorioallantoic membrane in vivo assay, survivin expression in tumor cells stimulated VEGF liberation and blood vessel formation [115].

In hepatocellular carcinoma HepG2 and SMMC-7721 cells, co-pretreatment with tunicamycin and melatonin potentiated the apoptotic effect of doxorubicin. Simultaneously, Akt activation by phosphorylation was elevated in tunicamycin treated cells but inhibited in the presence of melatonin. The downregulation of the PI3K/Akt pathway was associated with reduced levels of survivin [116]. In the same cell lines, the effect of melatonin on survivin was correlated with a reduced expression of COX-2, an inductor of angiogenesis, suggesting that melatonin inhibition of the COX-2/PI3K/Akt signaling pathway is the mechanism involved in the downregulation of survivin [117]. The ability of melatonin to downregulate survivin might have clinical implications. It has been described that co-treatment of MCF-7 cells with tamoxifen and melatonin-loaded in nanostructured lipid carriers significantly increased apoptosis accompanied by an increase in the pro-apoptotic Bid protein and a remarkable decrease in survivin expression [118].

In colorectal cancer Caco-2 cells, the combination of doxorubicin and melatonin was more efficient than each compound alone on cell proliferation, tumor spheroid formation, migration, and invasion. The expression of survivin, MMP-2, and MMP-9 was further decreased in response to both compounds [119].

NF-kB is constitutively activated in many human cancers. This family of transcription factors are often constitutively activated in cancer and contribute to tumor progression by regulating the expression of genes involved in cell proliferation, angiogenesis, and metastasis. Some genes upregulated by NF-kB, such as c-IAP1, c-IAP2, c-FLIP, Traf1, Traf2, A20, Bfl1/A1, and Bcl-xL, have anti-apoptotic effects. Other genes upregulated by NF-kB, such as VEGF, IL-8, uPA, and MMP9, are involved in angiogenesis [120].

The anticancer effect of melatonin on melanoma cells has been tested in combination with fisetin, a bio-flavonoid widely found in plants. The combination of both molecules promoted apoptosis and inhibited iNOS and COX-2 expression, impeded the translocation of p300 and NF-kB proteins to the nucleus, and prevented the binding of NF-kB on COX-2 promoter, thus preventing the activation of the PI3K/Akt signaling pathway [121]. Similar results have been described in the triple negative human breast carcinoma MDA-MB-231 cell line, when melatonin was tested in combination with tunicamycin. Melatonin abrogated both tunicamycin-induced COX-2 expression and NF-kB activity, preventing p65 translocation to the nucleus and p38 MAPK activation [122].

Melatonin and berberine, a plant derived molecule, effectively inhibit lung cancer cells proliferation and migration. Both compounds cooperated promoting apoptosis. At molecular level, they inhibited telomerase, translocation of NF-kB to the nucleus, and their binding to the COX-2 promoter. Additionally, melatonin enhanced the inhibition exerted by berberine on Akt and ERK activation [78].

A combination of melatonin and cisplatin has been evaluated in hepatocellular carcinoma cells. Melatonin enhanced cisplatin-mediated apoptosis. Concerning angiogenesis, once more, melatonin abolished the nuclear translocation of NF-kB p50/p65 proteins, blocked the recruitment of p65 to the COX-2 promoter, thus leading to inhibition of COX-2 expression, demonstrating that melatonin might contribute to cisplatin sensitization and stimulate cisplatin-mediated cell growth suppression, migration, angiogenesis, and metastasis via inactivation of the NFkB/COX-2 signaling pathway in hepatocarcinoma cells [123]. In bladder cancer, melatonin and curcumin, a natural polyphenolic compound, proved to be effective by suppressing the nuclear translocation of NF-kB and their binding to the COX-2 promoter. This effect of the co-treatment was a consequence of IKKb activity inhibition [124].

In vivo, melatonin showed therapeutic advantages in a model of nude mice bearing lung metastasis of gastric cancer. Those animals treated with IL-1β and the pineal hormone developed a lower number of lung metastases, and the size of the nodules were smaller. IL-1β had some deleterious effects, among them, increased levels of Snail or decreased levels of E-cadherin, upregulation of MMP-2 and MMP-9 expression, and activation of NF-kB. The pineal hormone effectively reverted all these non-desired IL-1β actions [125].

Although increased angiogenesis has been described in hematologic cancers, its role in leukemias is still not well characterized. It seems that PIGF (placenta growth factor) blockade, a VEGF family member, might have therapeutic potential [126]. In a MLL-rearranged leukemia xenograft mouse model, melatonin effectively suppressed the NF-kB/COX-2 signaling pathway in vitro and in vivo [127].

Cadherin T (cadherin 13), encoded by the CDH13 gene, is a member of cadherin superfamily; in ductal carcinoma in situ and adjacent invasive ductal carcinoma breast cancer patients, aberrant methylation of the CDH13 promoter was a frequent early event. Its loss has been associated with tumor malignancy, invasiveness, and metastasis [128]. In the estrogen responsive MCF-7 breast cancer cell line, docetaxel treatment causes several unexpected undesirable effects on gene expression. The expression of TP53, CDKN1A (p53 and p21, involved in control of cell cycle progression), and CDH13 (involved in angiogenesis) were reduced in the cells treated with the taxane. Interestingly, melatonin neutralized the adverse effect of docetaxel, leading to an enhanced expression of these tumor suppressor genes, key regulators of cell cycle, and inhibitors of tumor progression [4].

Sorafenib is an inhibitor of multiples kinases, blocking the activation of VEGFR2, PDGFR, c-Kit receptors, b-RAF, and p38, and was the first anti-angiogenic agent firstly approved for hepatocarcinoma treatment; however, its efficacy is rather modest and sustained sorafenib treatment leads to hypoxia-mediated drug resistance [129]. In hepatocellular carcinoma Hep3B cells, melatonin enhanced sorafenib cytotoxic effects and helped to overcome the resistance mechanisms mediated by hypoxia. At pharmacological concentrations (2 mM), the pineal hormone potentiated the effects of sorafenib on Hep3B cells grown under hypoxia. HIF-1α was downregulated by melatonin. Additionally, melatonin and sorafenib co-treated cells showed a reduced expression of the HIF-1α-mitophagy targets, an impaired autophagosome formation, and subsequent mitochondria and lysosomes colocalization, suggesting that melatonin might improve the Hep3B sensitivity to sorafenib, downregulating HIF-1α translation, thus blocking the cytoprotective mitophagy induced by the microenvironment in hypoxic conditions [130]. In pancreatic ductal adenocarcinoma, probably the most lethal cancer with poor prognosis, melatonin synergized with sorafenib to inhibit the progression of tumor growth, both in vitro and in vivo. The effect might be explained by a blockade of PDGFR-β/STAT3 signaling pathway, accompanied by mitochondrial dysfunction and an increase in the apoptosis rate [131].

Finally, the anti-angiogenic molecule has been tested, in combination with melatonin, in acute myeloid leukemia cells. The synergistic actions of the pineal hormone combined with sorafenib against leukemia bearing the FLT3/ITD mutation were tested, both ex vivo and in vivo. The results showed that melatonin significantly potentiated the cytotoxicity induced by sorafenib in acute myeloid cells with FLT3/ITD through redox modification. Additionally, the combined action of sorafenib and melatonin was highly effective in MV4-11 xenografts and might help to overcome chemoresistance in patients bearing the FLT3/ITD mutation [132].

## 5. Melatonin Antiangiogenic Actions and Radiation Therapy

### 5.1. Antiangiogenic Actions and Radiation Therapy

Radiosensitizers are molecules able to increase the effects of radiotherapy in tumor cells [133,134], including different compounds, such as antioxidants or corticosteroids [135,136], that may show undesirable effects such as high toxicity [137]. Oxygen acts as a potent radiosensitizer since the generation of ROS directly correlates with tumor oxygenation [138].

It is widely assumed that radiation stimulates angiogenic processes through upregulation of VEGF production and survival factors [139,140], thus increasing tumor cell proliferation. For this reason, new strategies, such as a combined use of antiangiogenic therapy and radiation, have been tested in different tumor cell lines and preclinical models [138,141], indicating that antiangiogenic agents can enhance the therapeutic effects of radiotherapy through increasing tumor oxygenation [142,143] and restoring the dysfunctional tumor vasculature [144]. Among these compounds are angiostatin, anti-VEGF/VEGFR [145,146], EGFR inhibitors [147], COX-2 inhibitors, or tyrosine kinase inhibitors (TNP-470) [138]. In clinical trials with patients diagnosed of pancreatic cancer, bevacizumab combined with radiotherapy showed an important reduction of the tumor dissemination and increased the median survival up to 12 months [148]. Similar effects were reported in rectal cancer [149]. However, these promising outcomes in clinical practice, more recent trials are not so conclusive [143,150]. Thus, it is necessary to further explore new combinations of antiangiogenic compounds able to interfere with the angiogenic process on the tumor cells.

### 5.2. Melatonin as an Antiangiogenic Agent: Synergistic Effects with Ionizing Radiation

Melatonin is widely considered as a good radioprotector diminishing radiation-induced DNA damage [137,151]. In addition, the pineal hormone has been proposed as a radiosensitizer, increasing the oncostatic effects of radiation on tumor cells. In breast cancer, melatonin exerts its radioprotective effects through different mechanisms, such as reduction of DNA repair capacity [152], induction of aerobic metabolism leading to mitochondrial ROS generation, apoptosis and autophagy activation [153], modulation of estrogen biosynthesis [154], or upregulation of p53, thus increasing apoptosis in tumor cells. Moreover, melatonin exerts a wide range of antiangiogenic actions in breast cancer cells [4,22,23,155]. Since radiotherapy is commonly associated with an increase in inflammation and activation of angiogenic pathways, other possible mechanisms proposed to explain the indolamine radiosensitizer effect is its ability to modulate different steps of the angiogenic process, thus decreasing inflammation and tumor growth [137,156].

**Figure 2:**
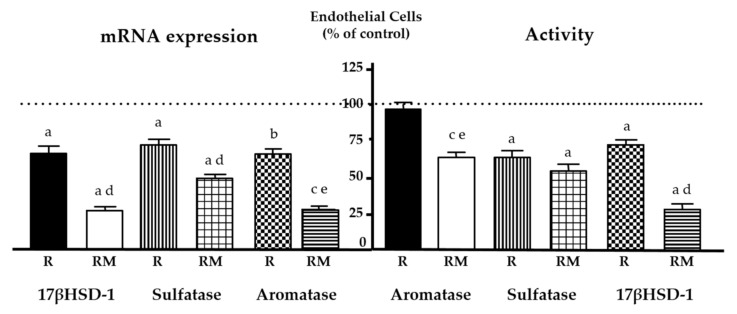
Effects of melatonin on radiation-induced changes on proteins involved in estrogen biosynthesis. Melatonin pretreatment (1 mM) before radiation (RM) increased the inhibitory effect induced by radiation (R) on aromatase, sulfatase, and 17βHSD-1 (17β hydroxysteroid dehydrogenase type 1) activity and expression on HUVEC endothelial cells. Data are expressed as the percentage of the control non-radiated group (mean ± SEM). (a), *p* < 0.001 vs. control non-radiated cells; (b), *p* < 0.01 vs. control non-radiated cells; (c), *p* < 0.05 vs. control non-radiated cells; (d), *p* < 0.001 vs. radiated cells; (e), *p* < 0.05 vs. radiated cells. Modified from González-González et al. [159].

In breast cancer, tumor microenvironment contributes to estrogen synthesis, thus affecting the tumor growth and the response to treatment [157,158]. Recently, it has been established that in co-cultures of human endothelial cells (HUVEC) and breast cancer cells melatonin increased the inhibitory effect of radiation on estrogen biosynthesis enzymes, aromatase, sulfatase, and 17β-HSD1 (Figure 2) [159]. Additionally, the indolamine potentiated the radiation effects on cell proliferation, tubular network formation, and migration, decreasing pro-angiogenic genes expression. Furthermore, melatonin counteracted the stimulatory effects of radiation on endothelial cell permeability through regulating the internalization of VE-cadherin and inhibited AKT/ERK signaling pathways [159]. Finally, in a chick chorioallantoic membrane (in vivo model of angiogenesis), melatonin was able to potentiate the inhibitory effects of radiation on the vascular area formation. In human breast fibroblasts, melatonin potentiated the oncostatic effect of radiation by decreasing aromatase expression and activity and inhibiting COX enzymes. Besides that, the indolamine counteracted the inhibitory effect of radiation on the pre-adipocytes differentiation by increasing the number of mature adipocytes [160], thus decreasing estrogen biosynthesis. The main targets of melatonin in radiated models are compiled in Table 1 [159,160].

The role of melatonin as an adjuvant treatment to ionizing radiation on human clinical trials has not been deeply assessed. In patients diagnosed with glioblastoma receiving melatonin associated to radiation, an increase in the 1-year survival rate and an improvement in the quality of life has been reported [161]. However, in a similar phase II study in patients with brain metastases, this positive effect could not be demonstrated [162].

## 6. Clinical Trials with Melatonin as Sensitizer to Other Antitumor Treatments

Some clinical trials have studied the action of melatonin on different tumor types, either alone or in combination with other anticancer treatments (Table 2). In its pioneer first clinical trial in 1991, Lissoni et al. [163] studied the effect of melatonin, both in tumor proliferation and in the patient’s quality of life, when all other therapeutic possibilities had been exhausted. They enrolled 54 patients, who were diagnosed with different solid tumors with distant metastases, without response to antitumor treatment. Lissoni’s group described that treatment with melatonin controlled, in a high percentage of patients, tumor progression and improved the quality of life [163]. One year later, the same group tested the combination of melatonin with interleukin-2 (IL-2) in patients with advanced non-small cell lung cancer [164].

Grimm et al. [165] had confirmed the antitumor immune response of IL-2. Lissoni et al. described that 20% of patients achieved a partial response to disease progression and 50% obtained tumor control, with a mean duration of 3.5 months. At the end of the treatment with melatonin plus IL-2, the number of T lymphocytes, NK cells, CD25, and eosinophils, had increased compared to the beginning of the trial. The results obtained from the combination of melatonin plus IL-2 as an immunostimulatory treatment in non-small cell lung cancer is well tolerated, as effective as chemotherapy, but does not damage the immune system [164]. Later, in 1996, it was also studied the possible synergy between melatonin and radiation therapy in patients diagnosed with glioblastoma. A dose of irradiation of 46 Gy (holocranial) + 14 Gy (directly to the tumor) was administered in doses of 2 Gy/day and patients also received 20 mg/day melatonin administered in the evening [161]. Thus, the administration of melatonin with radiotherapy in patients with glioblastoma increased the efficacy of radiation, prolonging its survival, and decreased the side effects produced by the radiation in these patients [161].

Dr. Lissoni conducted a new study in 70 patients diagnosed with non-small cell lung cancer and analyzed the impact of a therapy that combines melatonin with chemotherapy compared to a treatment based solely on chemotherapy as the first line of treatment [166]. The tumor regression rate achieved was higher in the double therapy group than in the group treated with chemotherapy alone, with a mean response duration of 9 months and 6 months, respectively.

Furthermore, the 1-year survival rate was higher also in the group that included melatonin in their treatment and the adverse effects produced by chemotherapy were less in the melatonin treated group [166]. Based on previous results, Lissoni et al., in 1999, analyzed the administration of melatonin with different chemotherapeutics (cisplatin + etopoxide, gemcitabine, doxorubicin, paclitaxel, mitoxantrone, 5-fluorouracil + folinic acid, 5-fluorouracil + cisplatin) in 250 patients with the diagnosis of distant metastases in non-small cell lung cancer, breast cancer, gastro-intestinal tract tumors, or head and neck cancers. The administration of melatonin induced a partial response in 29% of patients and a 5% of complete response compared to the group treated only with the chemotherapeutics, which obtained a partial response in 15% of patients and 0 of complete response. Thus, the tumor regression rate was significantly higher in patients also treated with melatonin than those who received chemotherapy alone. Additionally, survival at 1 year also was higher in the group treated with melatonin compared to the other [167]. Melatonin treatment helps to control the cellular damage imparted by TACE and to improve liver function, quality of life, and survival. The efficacy and survival rates were higher in the group with double therapy than with TACE alone [168]. It has been well documented that transcatheter arterial chemoembolization (TACE) induces hypoxia, and as a result, an increase in HIF-1, HIF-2, and VEGF [169]. Since angiogenesis will help residual cancer cells to survive, combination of TACE and angiogenesis inhibitors, such as sorafenib, would probably prevent the spread of hepatocellular carcinoma [170]. In a new study by Lissoni et al., a combination of melatonin and chemotherapy (cisplatin and etoposide) in patients diagnosed with non-small cell lung cancer was tested [171]. The tumor regression rate and survival obtained were, again, higher in patients who received dual therapy. Melatonin improved the effects of irinotecan in colorectal cancer patients whose disease progressed after treatment with 5-fluorouracil [172]. In other clinical trial, including 370 patients diagnosed with advanced cancer, the response rate and two-year survival was considerably higher in the group treated with chemotherapy and melatonin compared to the group treated only with the chemotherapeutics, whereas the adverse side effects (thrombocytopenia, neurotoxicity, asthenia, and cachexia) were reduced [173]. In lung adenocarcinoma, the combination of melatonin with somatostatine, retinoids, vitamin D, bromocriptine, and cyclophosphamide reduced respiratory and general symptoms, as well as decreased side effects [174]. In addition, in non-small cell lung cancer, the association of melatonin with different chemotherapy regimens induced a better assessment of health-related quality of life, but it did not modify survival and side effects [175]. In rectal or cervical cancer patients treated with pelvic radiation therapy, melatonin enhanced the antitumor action and efficacy of IL-2, amplified lymphocyte proliferation, and reduced the number of CD4s [176]. The failure of many antitumor treatments is caused by a decrease in the patient’s immune response, often related to an unexpected increase in the number of CD4 lymphocytes, the main cells in the suppression of this immunity through the release of immunosuppressive cytokines [177,178]. Thus, the inhibition of these CD4 lymphocytes represent an antitumor immunotherapy mechanism [14]. Regarding melatonin, the main immunological effects against cancer are: enhancement of IL-2 secretion [179] and inhibition of macrophages suppressive response [180].

In 2005, a meta-analysis of 10 clinical trials with melatonin alone or combined with chemo or radiotherapy review of randomized controlled was carried out. Melatonin exerted a positive effect on patients with advanced cancer, both in survival rates, disease progression, and in relation to side effects [181]. Furthermore, the beneficial effect was independent of tumor type [179]. In 2012, Seely et al. performed a meta-analysis including data from 21 clinical trials of solid tumors treated and concluded that melatonin decreased 1-year mortality, improved outcomes of complete and partial response, and ameliorated chemotherapy side effects [182].

In patients with head and neck cancer, radiation can cause mucositis, probable produced by oxygen free radicals resulting from radiotherapy. Melatonin could be a good therapeutic method based on its antioxidant effect [183]. In another double-blind clinical trial, melatonin diminished the severity of oral mucositis after radiotherapy, as well as reduced oral lesions and the pain produced by them in patients [184]. Melatonin also showed a protector effect in rectal cancer patients treated with radiotherapy [185]. Recently, a clinical trial investigated the effect of melatonin as adjuvant to chemotherapy in squamous cell carcinoma showed no significant effect on miR-210, CD44, and HIF-1α expression [186].

As a conclusion of these clinical trials, melatonin may safely increase 1-year survival and response rates when added to several forms of standard cancer care and may also alleviate the toxicity related to chemotherapy and improve cancer-related symptoms, as well. However, there are two limitations in some of these studies: many of the clinical trials were conducted by the same group of investigators and the lack of blinding. Independently conducted well-designed trials are needed to confirm these findings. Regarding the use of melatonin as an adjuvant to radiotherapy, there are few clinical trials with a low number of patients studied. In clinical trials on the use of melatonin as an adjunct to other antitumor treatments, none of them studied the antiangiogenic actions of melatonin as mediators of the effects found.

## 7. Conclusions

**Figure 3:**
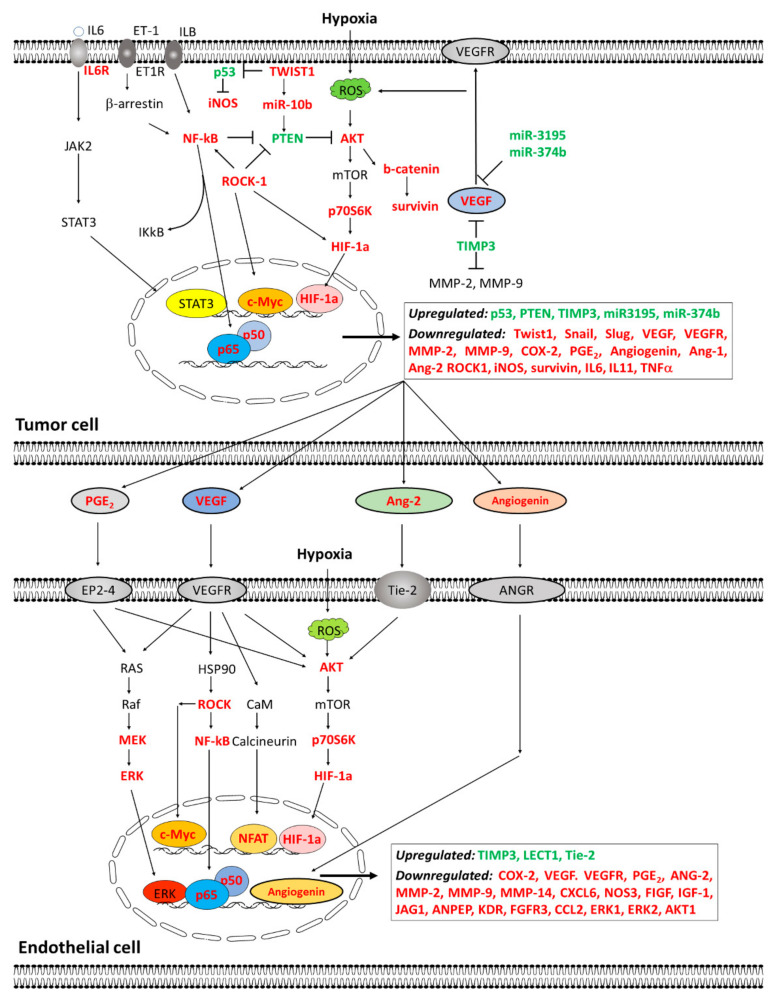
Proposed model of the pathways modulated by melatonin either in the tumor cell or the endothelial cell. In tumor cells, melatonin reverts the induction by chemotherapy agents of numerous EMT, pro-angiogenic, and pro-metastatic factors: Twist1, Snail, Slug, Survivin, miR-10b, COX-2, PGE2, VEGF, VEGFR, MMP2, MMP9, angiogenin, Ang-1, Ang-2, IGF-1R, ROCK-1, and HIF-1α, whereas it stimulates tumor suppressor genes and miRNAs, such as PTEN, p53, TIMP3, miR-3195, and miR374b. Mechanistically, melatonin inhibits the AKT, NF-kB, JAK2/STAT3, or ROCK-1 signaling pathways, suppresses STAT3 activation and NF-kB nuclear translocation, and it interferes with the tumor/endothelial cell communication by diminishing PGE2, VEGF, Ang-2, and Angiogenin release. In endothelial cells, melatonin inhibits the AKT, NK-kB, ERK, Rock-1, and calcineurin/NFAT pathways, and it diminishes NF-kB, ERK, and HIF-1α nuclear translocation, therefore reducing the expression of pro-angiogenic factors, such as COX-2, VEGF, VEGFR, PGE2, ANG-2, MMP-2, MMP-9, MMP-14, CXCL6, NOS3, FIGF, IGF-1, IGF-1R, JAG1, ANPEP, KDR, FGFR3, CCL2, ERK1, ERK2, and AKT1, and increasing anti-angiogenic proteins, such as TIMP-3, LECT-1, or Tie-2.

Numerous studies have described that melatonin has inhibitory effects on tumor growth. Among the mechanisms involved in the anti-cancer effects of melatonin are antiangiogenic actions. The inhibitory effects of melatonin on angiogenesis process are exerted at different levels, by regulating different proteins involved in the formation of new vessels. VEGF and HIF-1α seem like the main targets of melatonin. However, melatonin, unlike other inhibitors of angiogenesis, exerts actions on different proteins involved in the formation of new vessels by modulating the expression of different angiogenesis-related genes. Angiostatic properties of melatonin also include mechanisms, such as inhibition of endothelial cell proliferation, survival, migration, invasion, cell permeability, budding, or tube formation. Although the exact mechanisms by which melatonin inhibits angiogenesis are not fully described, this indolamine exerts inhibitory effects on mediators for several signal transduction pathways involved in cellular processes related with vascular remodeling, such as proliferation, survival, migration, and metabolism. Figure 3 summarizes upregulated and downregulated proteins by melatonin in endothelial cells and tumor cells.

In vitro and in vivo results suggest that combinations of melatonin with different antitumor drugs and chemotherapeutics result in improved efficacy of the treatments. Melatonin alone has been extensively documented as an effective inhibitor of proliferation, migration, and invasion. Many studies have linked melatonin to inhibition of VEGF and inactivation of HIF-1α meaning that melatonin neutralizes pro-angiogenic and potentiates antiangiogenic effects induced by chemotherapeutic agents or radiation, enhancing their antitumor effectiveness. At this moment, there are not many clinical trials testing the usefulness of melatonin and chemo- or radiotherapy association in humans. At present, in the ClinicalTrials.gov database, there are 60 clinical trials carried out on the use of melatonin in different types of cancer: breast cancer, colorectal cancer, lung, oral squamous cell carcinoma, melanoma, etc. Thirty-four of them are concluded, and eighteen are developing now. There are clinical trials in process that study the effects of melatonin in cancer patients on quality of life, appetite, sleep disorders, anxiety, depression, and fatigue. Four of them study the effects of melatonin as adjuvant to other antitumor treatments.

At this moment, there are two studies in process that investigate the administration of melatonin as adjuvant to radiotherapy. In summary, the efficacy of melatonin to sensitize cancer cells to chemotherapeutic agents or ionizing radiation makes it a molecule that can be effective as an adjuvant to these cancer treatments, and we reckon that more clinical trials combining melatonin and either chemotherapy or radiotherapy should be done at once.

## Figures and Tables

**Table 1:** Melatonin radiosensitizing effects as an antiangiogenic agent on endothelial cells.

**Changes in Angiogenesis related genes expression**	**Cytokines and angiogenic factors**	↓ Expression of *CCL2*	Immunoregulatory and inflammatory processes
		↓ Expression of *CXCL6*	Chemotactic and angiogenic properties
		↓ Expression of *ERK1/2, AKT1*	Cell growth and differentiation
		↓ Expression of *VEGFA, ANGPT2*	Angiogenesis and vascular development
	**Growth Factors and receptors**	↓ Expression of *FGFR3, IGF-1*	Breast cancer: migration and proliferation
		↓ Expression of *JAG1*	Hematopoiesis and angiogenesis
		↓ Expression of *TGFα*	Cell proliferation and differentiation
		↓ Expression of *KDR, ANPEP*	Angiogenesis and vascular development
	**Proteases and extracellular matrix molecules**	↓ Expression of *MMP14*	Cell growth, migration and tumor invasion
		↑ Expression of *TIMP1*	Degradation extracellular matrix and suppress proliferation
**Modulation of estrogen biosynthesis**		Decrease aromatase, sulfatase and 17β-HSD1 expression and activity	
		Inhibition of COX1/COX2 enzymes	
**Modulation of different steps of the angiogenic process**		Decrease vascular area (CAM assay)	
		Inhibition of the activation of p-AKT and p-ERK	
		↓ Permeability and VE-cadherin internalization	
		Inhibition of cell proliferation, migration, and tubular network	

*CCL2*, chemokine (C-C motif) ligand 2; *CXCL6*, chemokine (C-X-C motif) ligand 6 (granulocyte chemotactic protein 2); *ERK*, extracellular signal-regulated kinases; *AKT1*, V-akt murine thymoma viral oncogene homolog 1; *VEGFA*, vascular endothelial growth factor A; *ANGPT2*, angiopoietin 2; *FGFR3*, fibroblast growth factor receptor 3; *IGF-1*, insulin-like growth factor 1 (somatomedin C); *JAG1,* jagged canonical Notch ligand 1; *TGFα*, transforming growth factor, alpha; *KDR*, kinase insert domain receptor (a type III receptor tyrosine kinase); *ANPEP,* Alanyl (membrane) aminopeptidase; *MMP14,* matrix metallopeptidase 14 (membrane-inserted); *TIMP1*, TIMP metallopeptidase inhibitor 1; 17β-HSD1, 17β hydroxysteroid dehydrogenase type 1; COX1/COX2, cyclooxygenases type 1, 2; CAM, Chick Chorioallantoic Membrane.

**Table 2:** Clinical trials with melatonin as sensitizer to other antitumor treatments.

Tumor Type	Treatment	Melatonin Dose	Effects	Reference
Metastatic tumors	Chemotherapy	Induction 20 mg/day Then 10 mg/day	↓ Tumor progression	[163]
Advanced non-small cell lung cancer	Interleukin-2	10 mg/day starting 7 days before interleukin-2	50% Tumor control 20% Partial regression immunostimulatory effect	[164]
Glioblastoma	Radiotherapy	20 mg/day	↑ Survival ↓ Side effects	[161]
Non-small cell lung cancer	Cisplatin + Etoposide	20 mg/day	↑ Tumor regression ↑ Survival ↓ Side effects	[166]
Non-small cell lung cancer, breast cancer, gastrointestinal tract cancer, head and neck cancers	Cisplatin + Etoposide Gemcitabine Doxorubicin Paclitaxel Mitoxantrone 5-Fluorouracil + Folinic acid 5-Fluorouracil + Cisplatin	20 mg/day, starting 7 days before chemotherapy	↑ Tumor regression ↑ Complete response ↑ Survival	[167]
Advanced hepatocarcinoma	Transcatheter arterial chemoembolization	20 mg/day starting 7 days before	↑ Efficacy ↑ Survival Improve liver function	[168]
Non-small cell lung cancer	Cisplatin + Etoposide	20 mg/day starting 7 days before	↑ Tumor regression ↑ Survival	[169]
Advanced colorectal cancer	Irinotecan	20 mg/day	↑ Tumor regression	[170]
Different types of cancer	Several chemotherapeutic agents Radiotherapy	10 mg/day 20 mg/day 40 mg/day	↑ Tumor response ↑ Survival ↓ Side effects	[179]
Gastric cancer Colorectal cancerNon-small cell lung cancer	Cisplatin + Etoposide Cisplatin + Gemcitabine Oxaliplatin + Folinic acid +5-Fluorouracil Cisplatin + Etoposide+ leucovorin + 5-Fluorouracil 5-Fluorouracil + Folinic acid	20 mg/day	↑ Tumor response ↑ Survival ↓ Side effects	[171]
Lung cancer	Somatostatine, Retinoids, Vitamin D, Bromocriptine, Cyclophosphamide	20 mg/day	↓ Side effects ↓ Respiratory and general symptoms	[172]
Rectal cancer Cervical cancer	Pelvic radiation IL-2	20 mg/day	↑ Efficacy IL-2 ↑ Lymphocyte proliferation ↓ Number CD4s	[174]
Different types of cancer	Several chemotherapeutic agents	20 mg/day 40 mg/day	↑ Tumor response ↑ Survival ↓ Side effects	[180]
Non-small cell lung cancer	Chemotherapy regimens	10 mg/day 20 mg/day	Better assessment of health-related quality of life	[173]
Rectal cancer	Radiotherapy	20 mg/day	↓ Side effects in blood	[183]
Head and neck cancer	Radiotherapy	20 mg/day	↓ Mucositis ↓ Oral lesions	[182]
Oral squamous cell carcinoma	Taxane, Cisplatin, 5-Fluorouracil	20 mg/day	↓ miR-210 and CD44 but not significant	[184]

## Data Availability

No new data were created or analyzed in this study. Data sharing is not applicable to this article.
